# Effect of Aerobics Exercise on Self-Esteem in Iranian Female Adolescents Covered by Welfare Organization

**DOI:** 10.1155/2014/456483

**Published:** 2014-12-25

**Authors:** Marzieh Hasanpour, Mansooreh Tabatabaei, Mousa Alavi, Vahid Zolaktaf

**Affiliations:** ^1^Nursing and Midwifery Care Research Center, Pediatric and Neonatal Nursing Education Department, Faculty of Nursing and Midwifery, Isfahan University of Medical Sciences, Isfahan 81746-73461, Iran; ^2^Pediatric and Neonatal Nursing Education Department, Faculty of Nursing and Midwifery, Tehran University of Medical Sciences, Tehran, Iran; ^3^Student Research Center, Nursing and Midwifery School, Isfahan University of Medical Sciences, Isfahan, Iran; ^4^Nursing and Midwifery Care Research Center, Psychiatric Nursing Department, Faculty of Nursing and Midwifery, Isfahan University of Medical Sciences, Isfahan, Iran; ^5^Department of Exercise Rehab, Faculty of Exercise Sciences, University of Isfahan, Isfahan 81744, Iran

## Abstract

*Introduction*. Deprivation of parents might decrease self-esteem (SE) and result in affective and social incompatibility. In this randomized control trial, we examined the effect of aerobics exercise on SE among female adolescents living with no natural family.* Materials and Methods*. The sample consisted of all female adolescents aged 13 to 19 years (*n*: 72) who were covered by Isfahan Welfare organization. Participants were assigned into intervention and control groups by matched random sampling. Intervention included 8 weeks of aerobics exercise. Coppersmith SE inventory was administered before and after intervention as well as after one month follow-up.* Results*. No significant difference was seen between pre-SE scores of intervention (32.7 ± 8.4) and control (33.0 ± 6.7) groups (*t* = .16, *P* = .87). A significant difference was obtained in post-SE scores (40.2 ± 5.7 versus 34.7 ± 6.8, *t* = 3.58, *P* = .001) and in one month follow-up scores (36.4 ± 5.2 versus 33.0 ± 5.2, *t* = 2.25, *P* = .03).* Discussion*. The results demonstrated a low level of pre-SE in both groups. However, a significant improvement was seen in posttest of intervention group which persisted even one month after intervention. It supports the use of aerobics for female adolescents deprived from family life.

## 1. Introduction

One of the basic needs of human is a sense of self-worth or self-esteem that affects their growth, development, and identity. Self-esteem has internal and external aspects. The external aspect enables people to behave in such a way to appear self-confident in view of the outside world, and internal aspect gives people a sense of being good. Each consists of a number of indicators. Internal aspect includes self-love, self-recognition, having clear goals, and positive thinking, and external aspect includes communication with others, self-belief, appearance in community, and control of emotions. Nurturing and development of these indicators begin at home and especially with parents [1]. Self-esteem is based on kindness and love of children, like a strong foundation for the house [2, 3]. Low self-esteem causes behavioral and emotional disorders and may lead to antisocial behaviors [4–6]. According to Islamic literature, underdeveloped self-esteem creates a sense of inferiority, which leads to further psychological problems including lies, arrogance and selfishness, aggression, and ambition [7, 8]. On the contrary, people with high self-esteem are academically successful and have greater social participation, attach more importance to exercise, and enjoy greater social and emotional compatibility, which plays a stronger role in adolescents [9, 10]. Although formation and development of self-esteem continue throughout a person's life, growth and development appear to dominate and play a more significant role in some periods, including adolescence [3].

Today, with the population of over 3 billion, ratio of adolescents to the world population is greater than any other time before. In Iran, according to the latest census, population of this prosperous generation exceeds 17 million [11]. Adolescence years are considered a prominent stage in a person's social and mental development. Self-esteem and identity, which affect each other, are components of this period. Identity is a distinction that an individual considers to have in comparison with others and depends upon mental, social, and physical structure. Finding identity in adolescence is much more highlighted due to a series of new psychological, mental, biological, and social changes. Finding the right identity leads to adolescent's better formation of self-esteem, which provides the basis for growth and improvement, whereas, with inappropriate formation of identity, an individual will be frustrated and lack self-esteem, and consequently, they will be isolated instead of interacting with others and will have confused and vague personality instead of self-awareness and established identity [12, 13].

There are different opinions about the equality of self-esteem among sexes [14]. For instance, young girls have greater problems in maintaining self-esteem than boys and often rate their own self-esteem poorly until adulthood [13]. On the contrary, Ucklechi et al. (2010) reported that self-esteem of 358 female adolescents was high in Sabzevar [15].

Family, school, and society are among influential factors in social and mental development and formation of self-esteem. Family, as an important factor, plays a significant role in upbringing of children, especially in childhood and adolescence. Within the family, a key role is played by mothers, with a significant role in children's growth and development [16]. Adolescents deprived of this source of support, especially in childhood, are adversely affected. According to a study conducted in Iran on 360 orphan and nonorphan children with the aim to assess the prevalence of psychosocial problems in orphans, stress, anxiety, intelligence, social growth, and self-esteem were measured. The results revealed significantly poor levels of social growth, intelligence, and self-esteem and greater levels of stress among these children compared to children with parents and also equal levels of self-esteem in both sexes in orphan children [16].

According to statistics published by Iran's Welfare Organization in 2012, a total of 21 thousand children are under the umbrella of this organization, of whom 11500 are in foster families and 9500 in children and adolescents' care homes of Welfare Organization. Accordingly, parents of 3% of these children are missing, 21% have disqualified parents, 16% have divorced parents, 9% have their parents in jail, 9% have been abused by their parents, 20% have deceased parents, and 3% have parents with incurable diseases, and thus they have been left with organization's children and adolescents care homes, and, unfortunately, their numbers increase by 10% every year [11, 17].

In Isfahan province, the number of homeless children and adolescents in 2012 was reported 1200, of whom 750 were taken in by families and relatives, and 450 are kept in welfare homes. In Iran, there are 476 such centers for housing these children, of which 125 are public and 351 private, and out of these 35 centers are located in Isfahan Province, of which 11 are in Isfahan City. Of the 450 children covered by welfare, 280 are 12- to 19-year-old adolescents, 180 are girls and the rest are boys [18].

Adolescents in boarding centers have difficulty in establishing interpersonal relationships, which extends from inside the center to the network of peers. Frequent relocations in different centers make it difficult to maintain and to have consistent relationships. Furthermore, adolescents often argue that they are stigmatized by their peers for living in these centers. Adolescents suffer low self-esteem in these centers due to lack of a sense of belonging, damaged identity trepan confusion, and lack of long-term relationships [15, 19].

Other influential factors in increasing self-esteem include establishment of social interactions with peers, learning social skills, leisure time, exercise, music, and motivation. Research shows that regular exercise leads to physical health as well as mental health, dealing with problems, depression, and increased self-understanding and self-esteem in men and women [20–22]. Exercise, which covers dance and activities done without using devices [23], affects memory, increases longevity, reduces risk of various diseases, and increases self-esteem [24]. According to research, dancing or doing aerobics 3 times per week reduces anxiety and depression in people [25, 26].

Based on the above, it can be inferred that self-esteem is an important psychological issue in human, in adolescents, depending on their circumstances. For instance, adolescents that live in pseudofamilies and boarding centers not only face physical and emotional problems, but have low self-esteem. Most studies have shown that girls have lower self-esteem compared to boys, while other studies state the opposite. However, it should be noted that self-esteem depends on social, upbringing, cultural, and also personal circumstances. Some studies have used a variety of methods such as psychotherapy, group therapy, and lifestyle changes in order to elevate self-esteem. Yet, there are very few studies on the effect of aerobic exercise on self-esteem. Ultimately, one of the most vulnerable groups in the community is the group of orphan children and adolescents that are deprived of parental care and are faced with such problems as having to be cared for by different families for different periods of time, with frequent displacements and confusion, which all adversely affect their self-esteem. The process is impaired, especially in adolescence that is a period of transition and development of identity. Furthermore, with researchers' personal experiences and seeing these children's problems, including low self-esteem and their later problems, priority of dealing with orphan adolescents' self-esteem by the provincial welfare officer is recognized as an important challenge for these centers. Also, given that most studies have rated girls' self-esteem less than boys' [13, 15, 19] and since adolescents' mental and social health are responsibility of pediatric nurses, there is a need for intervention to improve and elevate self-esteem of adolescent girls covered by Welfare Organization. Thus, research team decided to conduct a quantitative interventional study with the aim to assess the effect of aerobic exercise on self-esteem of Iranian adolescent girls covered by the Welfare Organization.

## 2. Materials and Methods

This is an experimental, quantitative study, with random division of subjects into case (exercise) and control groups. This study comprised 3 stages of pretest and the first and the second posttests in order to assess the effect of independent variable of aerobic exercise on dependent variable of self-esteem in adolescent girls. The second posttest was conducted after a month because of the lasting effect of exercise.

In this study, subjects were selected by census and were divided into intervention and control groups using SPSS software. Permissions were obtained from the Research Deputy of School of Midwifery and Nursing, Ethics Committee of Isfahan University of Medical Sciences (Project number 392088), and also from Isfahan Province Welfare Organization, and written consents were obtained from adolescent or their legal guardian (depending on legal age). Then, study objectives were explained and Coppersmith's self-esteem questionnaire was given to adolescents who were asked to complete it after careful study. Demographic and underlying information of study subjects were also collected through a questionnaire. A number of subjects were excluded due to various reasons including age outside 13 to 19 years, ongoing treatment for physical or mental problems, and more than 3 lie-detector responses, thus reducing the number of study subjects from 72 to 66. The reasons are the criteria for inclusion and exclusion. Study subjects were selected from 3 pseudofamily centers across Isfahan city and were randomly divided into intervention and control groups. The control group received no intervention at all, but the intervention group underwent aerobic exercises in pairs by the researcher and a physical education trainer (Master's student). On June 26 until August 22, 24 sessions of aerobic exercise were performed.

Intervention was performed for 8 weeks, 3 sessions per week and 60 minutes for each session (warming up, aerobics, and cooling down), warming up and cooling down took 10 minutes each, and aerobic exercise lasted 40 minutes. During warming up with stretch moves, music was played at a soft rhythm, which gathered pace with movements. Cooling down included stretch moves with slow rhythm. Music was so selected to use up to 60% to 80% of reserved hear rate in subjects. Subjects' heart rate during exercise was controlled through wrist pulse, and reserved heart rate (HRR) was calculated using Karvonen formula, as follows.

 Maximum heart rate = 220 − 20; for example, 220 − 20 = 200
 Resting heart rate = 70.
 Heart reserved rate = Resting heart rate − maximum heart rate; for example, 200 − 70 = 130.
 Lowest heart rate = resting heart rate + 60% × heart rate reserve; for example, 130 × 60% + 70 = 148.
 Highest heart rate = resting heart rate + 80% × heart rate reserve; for example, 130 × 80% + 70 = 174.
 Range of exercise heart rate = 148 to 174 beats per minute.

Subjects were trained by researcher to control their radial pulse before exercise and were asked to take and record their radial pulse on 3 successive days after waking up and before any movement, with insignificant differences, to record mean heart rest rate, which was between 67 and 80.

Following the last exercise session and a month after, self-esteem questionnaire was again made available to subjects in both groups to complete.

Study data were collected through a two-part questionnaire. The first part was about demographic details and the second about Coppersmith self-esteem inventory (CSEI). CSEI for adolescents consists of 58 items and 4 subscales including self (31 items), peers (5 items), family (8 items), academic (6 items), and, the remaining questions, the lie scale (8 items). To answer questions, score is from 0 to 50, which is as follows: score of 35 and down, very low self-esteem; score of 35 to 39, low self-esteem; score of 39 to 43, the average self-esteem; and score of 43 to 46, good self-esteem; and finally a score of 46 to 50, is considered a high self-esteem.

Since study subjects had no families, the term guardian (from pseudofamily centers) was used instead of family to resolve this problem, and validity of the questionnaire was reevaluated. CSEI was designed and developed by Smith et al. in 1976, and its retest coefficients were reported 88% after 5 weeks and 77% after 3 years. Internal consistency of this inventory was found by Ebrahimi-Ghavam (1982) on a sample of elementary school students in Ahwaz-Iran, and Cronbach's alpha was reported 82% [27].

In this study, items 5, 19, 26, 23, 47, and 54 of CSEI related to parents, home, and family, and since subjects were separated from their parents and lived in the welfare's pseudofamily centers, hence instead of terms relating to home and family, guardians and pseudofamily were used. Therefore, content validity of Coppersmith questionnaire and demographic details was reassessed by 15 expert faculty members in psychiatry, psychiatric-nursing, pediatric and family nursing, psychology, and pediatrician disciplines at Isfahan University of Medical Sciences, and in a joint meeting with members of research team comments from experts were examined and implemented, and content validity was determined. Reliability of questionnaire was assessed using internal consistency, and Cronbach's alpha was found 86%.

Descriptive and inferential statistics, that is, analysis of covariance (ANCOVA), were used to investigate the differences between the intervention and control groups. ANCOVA with baseline score as the covariate was used to test for the effect of the intervention (interaction effects between intervention/control groups). Initially, basic conditions and assumptions of ANCOVA such as continuous covariates that were not affected by treatment, linearity between the covariate and the dependent variable, equality of the regression slopes within group (*F*_1,62_ = 0.92, *P* = 0.34), and homogeneity of variance (*P* > 0.1) were met.

Chi-square and Mann-Whitney tests and independent *t*-tests were used to perform baseline comparisons between the intervention and control groups in terms of demographic and contextual variables. Data were analyzed with SPSS-16 software.

## 3. Results

In this study, participants included 66 orphan adolescent girls, aged 13 to 19 years, covered by Isfahan welfare centers that met study inclusion criteria. According to independent* t*-test and Mann-Whitney and Chi-square tests, distribution of study subjects in the two groups was the same in terms of demographic and underlying variables, including age, height, weight, reasons for welfare cover, length of stay at center and separation from parents, and education level of adolescent and guardian.

Mean self-esteem scores of girls covered by Isfahan welfare in intervention and control groups, before and after intervention and one month after intervention, are presented in Table 1. It can be seen that mean self-esteem score before intervention in intervention group was 32.73 ± 8.4 and in control group 33.03 ± 6.7. After intervention, mean self-esteem score significantly increased to 40.23 ± 5.7 in the intervention group and insignificantly to 34.66 ± 6.8 in the control group. One month after exercise, mean self-esteem score was 36.4 ± 5.2 in the intervention group and 33 ± 5.2 in the control group.

Independent *t*-test results (Table 1) showed insignificant differences in the mean self-esteem scores between the two groups before intervention (*P* = 0.87) but significant differences after intervention (*P* = 0.001). One month after intervention, results showed that despite the amount of time elapsed, the effects of aerobic exercise still persisted (*P* = 0.002).

**Figure 1:**
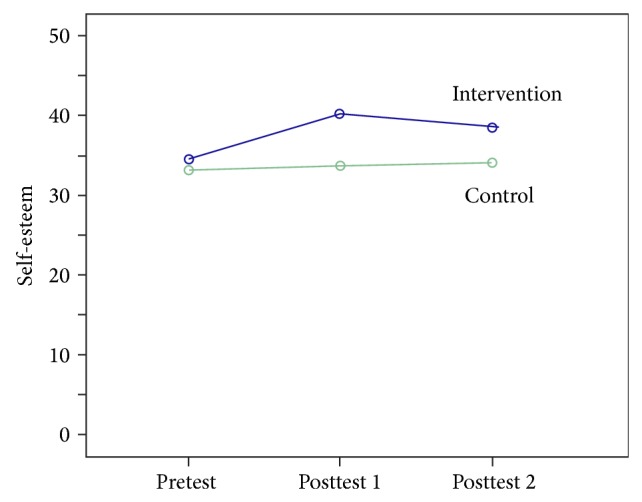


Figure 1 shows linear variations of self-esteem in the control and the intervention groups before, after, and one month after intervention. This figure shows control group's progress with a slow slope but intervention's progress with a fast slope. Also, one month after intervention, when aerobic exercise stopped, self-esteem score slightly reduced.

Average follow-up scores of the self-esteem (after intervention and one month after intervention) in both the intervention and control groups were calculated and treated as dependent variable in the ANCOVA (Table 1). The results (Table 2) were statistically significant (*F*_1,63_ = 18.6, *P* < 0.001). It confirmed the effect of the intervention on the outcome variable.

## 4. Discussion and Conclusion

The results indicate insignificant differences between the two groups in mean self-esteem scores before intervention. However, the difference between the two groups was significant after intervention. Mean self-esteem score of girls (in the range 0 to 50) was nearly the same and very low before intervention in the intervention group (32.73 ± 8.40) and the control (33.03 ± 7.6). After intervention, mean self-esteem score in the intervention group reached 40.23 ± 5.7, indicating moderate self-esteem, while, in the control group, the change was insignificant. In support of the present study results, a study in Africa by Foad with the aim to determine developmental and emotional differences among children in orphanage, conducted on 294 boys and girls with no parents or single parents, showed 21% depression, 45% anxiety, 61% developmental problems, and 23% self-esteem in these children, which are in line with the present study results. In his study, Fouad used Rosenberg questionnaire to assess self-esteem, which comprises 10 items on family and education, while, in the present study, modified CSEI was used for orphan children, which consists of 58 items with 4 subscales of family, academic, peers, and self [2].

The results of independent *t*-test showed insignificant differences between the two groups in mean self-esteem scores before intervention (*P* = 0.87), but significant differences were observed after intervention (*P* = 0.001). Mean self-esteem score immediately and one month after intervention in the intervention group was 38.3 ± 4.9 and 34.1 ± 5.7 in the control group. According to independent *t*-test results, the difference was significant between the two groups (*P* = 0.002).

The results of a study by Cohen et al. (2009) are in line with the present study results [28]. In his review article, Cohen investigated the effect of physical activity on psychological health in 31 articles. Quoting Rudolph (1998), Cohen states, “There is a relationship between cortisol and stress, and cortisol level balances with 30 minutes aerobic exercise with moderate intensity, and then reduces, which leads to a sense of well-being, health, increased self-esteem and reduced stress in the long term, and at moderate level, it improves self-acceptance, self-concept, and self-esteem, which confirms the present study results on the effect of aerobic exercise on self-esteem.”

The results of a study conducted in Turkey by Hakan Kolayis et al. (2011) on the effect of physical exercise on self-esteem and anxiety in children living in boarding centers with mean age of 11.4 years showed that these exercises affected children's anxiety and self-esteem. The present study examined the effect of aerobic exercise on self-esteem, while Kolayis addressed the effect of physical exercise on anxiety and self-esteem. Physical exercise comprises running, jogging, basketball, volleyball, and swimming and other exercises that do not require music and have not been specified. Moreover, study subjects were 25 girls and boys with mean age 11.4 years, which was different from the present study on 66 girls of 13 to 19 years old. The present study also addressed continued effect of aerobic exercise on self-esteem. However, generally, this study is in line with the present study in terms of positive effects of exercise.

The results of a study conducted to assess the effect of aerobic exercise and physical activity (swimming) on body image and physical self-perception in 55, 13- to 14-year-old girls in Britain, showed that variety of exercises have positive mental and psychological effects on humans, but the effects of aerobic exercise on physical perception and body image are greater than other exercises [4], while changes in self-esteem have not been reported (*P* = 0.1).

The results of another study conducted in 2009 by Saraf and Emami on the effects of aerobic exercise and Yuga on body image component in 19- to 25-year-old girls showed that aerobic exercise and Yuga did not have much effect on body image and rather affected coordination of body action and sporting competence [29]. In Burgess and Emami studies, different effects of aerobic exercise on body image are proposed, such that Emami does not consider aerobic effective on body image, while Burgess rates its effect positively. The difference may have been due to age of participants in Brook's study (13- to 14-year-old adolescents) compared to that in Emami's study (19- to 25-year-old girls). It should also be noted that subjects in Burgess study were children with parents [24].

According to results obtained that show low self-esteem in both groups before intervention and significant increase immediately and one month after intervention and considering welcoming of adolescent girls and center officials of aerobic exercise, which costs very little to implement, it is recommended that this exercise become part of weekly and formal programs of adolescents in welfare centers, so that, by increasing psychosocial health and self-esteem of this vulnerable group, a small step can be taken toward improving health of adolescent in the community.

The study was limited to adolescent girls covered by Isfahan Welfare Organization, which limits generalization of results to other populations. Thus, it is recommended that the effect of aerobic exercise on self-esteem and other psychological components be investigated in different age groups and in both sexes. Furthermore, it is recommended that the efficacy of other self-esteem increasing methods in children and adolescents covered by Welfare Organization be compared with the effect of aerobic exercise, so that, using these results, authorities and planners can provide the context for creating positive effects on mental health in those covered by Welfare Organization.

## Figures and Tables

**Table 1:** Comparison of self-esteem in adolescents between exercise groups (intervention) and control before, immediately after, and one month after intervention.

Groups	Statistical indicators							
	Pretest		Posttest 1		Posttest 2		Average post test	
	Mean	SD	Mean	SD	Mean	SD	Mean	SD
Intervention	32/73	8/4	40/23	5/7	36/40	5/2	38/30	4/9
Control	33/03	6/7	34/66	6/8	33	5/2	34/1	5/7
Independent samples *t*-test								
df	64		64		64		64	
*t*	−0/16		3/58		2/25		3/22	
*P* value	0/871		0/001		0/027		0/002	

**Table 2:** Analysis of covariance on posttest of the Iranian female adolescents' self-esteem as dependent variable.

Source	Mean square	df	*F*	Sig.
Pretest	753.02	1	44.42	0.000
Group	315.35	1	18.60	0.000
Error	16.95	63		
